# Beyond Hypomethylating Agents: Novel Therapies and Targeted Approaches

**DOI:** 10.46989/001c.142956

**Published:** 2025-08-07

**Authors:** Viviana Cortiana, Harshitha Vallabhaneni, Jenna Ghazal, Kennedy Itodo, Taha Kassim Dohadwala, Chandler Park, Yan Leyfman

**Affiliations:** 1 Department of Medical and Surgical Sciences (DIMEC), University of Bologna, 40126 Bologna, Italy; 2 Apollo Institute of Medical Sciences and Research, Hyderabad, India; 3 Kalamazoo Valley Community College, Kalamazoo, Michigan, USA; 4 Nigerian Institute Ffor Trypanosomiasis Research, Jos, Nigeria; 5 David Tvildiani Medical University, Tbilisi, Georgia; 6 Norton Cancer Institute, Louisville, KY, USA; 7 NewYork-Presbyterian Hospital, Brooklyn, NY, NY, USA

**Keywords:** Myelodysplastic Syndromes (MDS), Immune Checkpoint Inhibitors, Hypomethylating Agents (HMAs), Precision Medicine, Targeted Therapy, Hematologic Malignancies, Immunotherapy.

## Abstract

Myelodysplastic syndromes (MDS) represent a heterogeneous group of clonal hematopoietic diseases sharing ineffective hematopoiesis, cytopenias, and a high risk of evolution to acute myeloid leukemia (AML). The current MDS classification systems, such as the International Consensus Classification (ICC) and the WHO 2022 classification, have incorporated molecular and cytogenetic markers to improve the stratification of risk and guide therapy. However, treatment options for high-risk MDS (HR-MDS) remain limited, with hypomethylating agents (HMAs) providing only modest survival benefits. Emerging treatments such as immune checkpoint blockade and novel targeted therapies could further improve patient outcomes. While early excitement was significant, clinical trials of the immune checkpoint inhibitors (ICIs) ipilimumab and durvalumab have produced no definitive results, highlighting the need for better patient selection and combination regimens. Emerging drugs luspatercept and imetelstat have been suggested for lower-risk MDS (LR-MDS) by promoting transfusion independence and global hematologic response. In contrast, exploratory agents such as pevonedistat, magrolimab, and sabatolimab are under further investigation for HR-MDS. The future of MDS treatment currently addresses precision medicine, in which molecular characterization guides therapeutic options. Identification of predictive biomarkers for response to targeted therapies and immunotherapies is crucial to optimize patient outcomes. An integrated, patient-centered approach combining genomics, novel therapeutics, and immunomodulation is therefore essential to address the current needs in MDS management.

## Introduction

Myelodysplastic syndromes (MDS) are a group of myeloid neoplasms marked by ineffective hematopoiesis due to genetic and epigenetic alterations that impair cell cycling and proliferation.^1,2^ These disruptions affect hematopoietic stem and progenitor cells, resulting in multi-lineage cytopenias, dysplastic morphology, clonal genetic abnormalities, and a variable risk of progression to acute myeloid leukemia (AML).^1,3^

Approximately 20,000 new MDS cases are diagnosed annually in the United States, with a five-year survival rate of about 31%.^1^ Though uncommon in individuals under 50, the incidence rises significantly with age, with a median diagnosis age of 73–76 years.^1,3,4^ Older adults often face additional challenges, including comorbidities, polypharmacy, psychosocial stressors, and age-related physiological changes affecting renal and hepatic clearance, muscle mass, and organ function.^2^

Risk factors for MDS and its precursors—clonal hematopoiesis of indeterminate potential (CHIP) and clonal cytopenia of undetermined significance (CCUS)—include advanced age, male gender, smoking, prior cytotoxic therapy (i.e., secondary MDS), and environmental exposures such as ionizing radiation and industrial chemicals.^3–5^ Inherited syndromes like Fanconi anemia, Bloom syndrome, Down syndrome, and Diamond-Blackfan anemia also increase susceptibility, along with mutations in regulatory genes.^3–5^

As highlighted by Zeidan et al {1} during his keynote at MedNews Week, tailoring therapy in MDS is particularly complex due to its underlying genetic heterogeneity.^1^ Recurrent mutations occur in genes regulating DNA methylation (e.g., TET2, DNMT3A, ASXL1), RNA splicing (e.g., SF3B1, U2AF1, SRSF2), transcription (e.g., RUNX1, CEBPA, GATA2), chromatin modification (e.g., EZH2), signaling pathways (e.g., NRAS, KRAS, FLT3), and DNA repair.^6–8^ Diagnostic tools such as next-generation sequencing (NGS), Sanger sequencing, and single nucleotide polymorphism (SNP) arrays are critical for identifying these alterations.^6–8^

Another challenge in MDS management is its clinical heterogeneity. To address this, various classification systems have evolved. Earlier frameworks like the French-American-British (FAB) and WHO 2001/2016 relied on morphology and blast counts. In contrast, newer systems—such as the 2022 WHO update and the International Consensus Classification—incorporate molecular and cytogenetic markers (e.g., SF3B1, del(5q), and bi-allelic TP53 mutations) to enhance diagnostic precision and prognostication.^9,10^

Dr. Zeidan also underscored limitations in traditional prognostic models like the IPSS, WPSS, and MD Anderson risk scores, which often overlook individual comorbidities and biological context.^1^ These models provide general risk assessments but lack treatment-specific predictive power. For instance, patients with therapy-related MDS often experience poorer outcomes regardless of risk category.^1^ To address this, the IPSS-M—featuring a 31-gene panel—offers refined stratification and supports more personalized treatment planning throughout the disease course.^1^

**Figure 1.**
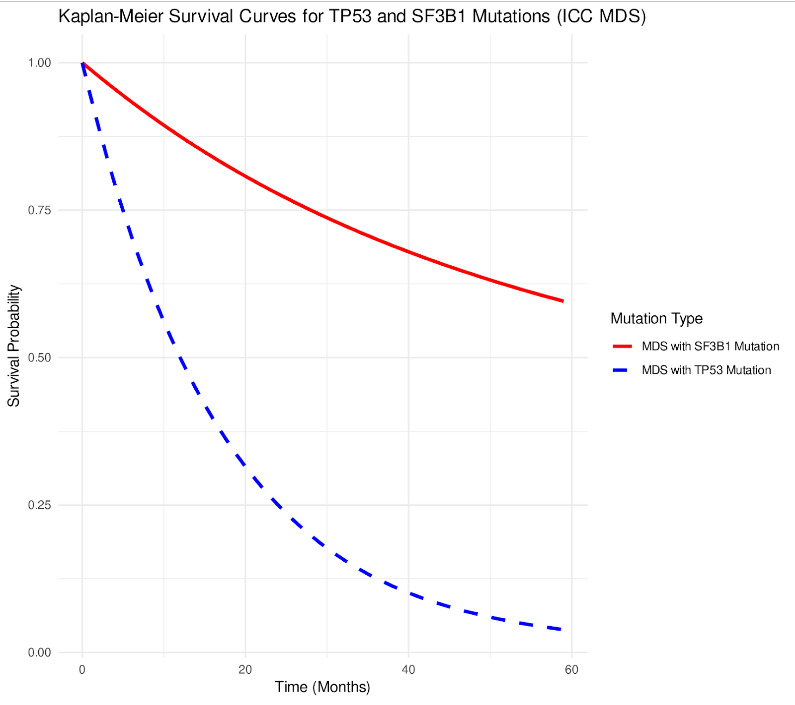
Kaplan-Meier survival curves illustrating significantly poorer survival outcomes for MDS patients with TP53 mutations compared to those with SF3B1 mutations, consistent with ICC-classified prognostic expectations.^11,12^

This commentary explores the current landscape of MDS, highlighting evolving classification systems, clinical challenges, and novel therapeutic strategies shaping the future of patient care (Figure 1).

## 2. Evolution of Response Criteria in MDS

The evolution of response criteria in MDS reflects a growing understanding of the disease’s pathophysiology, progressing from basic hematologic measures to more sophisticated molecular and genetic assessments. Early response evaluations primarily relied on clinical indicators such as peripheral blood counts and transfusion dependency.^13^ These initial criteria, focused on hemoglobin, neutrophil, and platelet levels, provided limited insight into the biological complexity and long-term prognosis of MDS.

A major milestone came with the introduction of the International Working Group (IWG) criteria in the early 2000s. These refined prior definitions by distinguishing complete and partial remissions and introducing categories for hematologic improvement, including reductions in transfusion dependence. However, these criteria remained rooted in clinical and hematologic parameters without incorporating molecular features.^14^

Limitations of the IWG criteria became particularly evident in high-risk MDS, where response measures sometimes overestimated therapeutic efficacy. The 2006 introduction of the “morphologic complete remission” (mCR) concept inadvertently led to misinterpretations of drug activity. To address these challenges, an international consortium proposed updated response criteria, now known as the IWG 2020 criteria.^1^ These updates included the elimination of unstable mCR definitions, a redefinition of complete remission with a lower hemoglobin threshold, and the introduction of intermediate categories for less-than-complete responses. These criteria are currently undergoing validation in clinical trials.

Concurrently, the integration of genetic and cytogenetic markers into response assessments marked a significant advancement. Somatic mutations and chromosomal abnormalities are now recognized as essential components of disease monitoring, enabling a more nuanced understanding of clonal evolution and therapeutic response.^15^

The increasing use of minimal residual disease (MRD) monitoring through next-generation sequencing (NGS) and flow cytometry has further enhanced response assessments. These technologies allow detection of molecular disease persistence that may not be reflected in peripheral counts, offering deeper insight into treatment efficacy and disease biology.^14^ This shift is especially relevant with the emergence of novel agents, including immune checkpoint inhibitors and targeted therapies, which may achieve durable molecular remissions without immediate hematologic improvements.

Looking forward, the response criteria in MDS are expected to continue evolving alongside therapeutic innovations and advances in molecular profiling. This dynamic landscape will enable more personalized treatment strategies based on individual genetic and disease characteristics, ultimately improving prognostic accuracy and patient outcomes.^16^

### Biomarkers Predictive of Treatment Response and Resistance

Recent large-scale studies have elucidated the dual role of somatic mutations in MDS, not only as markers of disease prognosis but also as predictors of therapeutic response. TP53 mutations, especially biallelic (“multi-hit”) events and high variant allele frequency (VAF), represent the most adverse molecular biomarker in MDS. A pivotal analysis of over 7,000 MDS patients revealed that multi-hit TP53 mutations conferred a hazard ratio (HR) of approximately 3.7 for overall survival (OS) compared to monoallelic TP53 mutations (median OS: 8.7 versus 2.5 years; HR = 3.7; 95% CI: 2.7–5.0; p < 0.001).^17^ Moreover, TP53 mutations are strongly associated with complex karyotypes, primary resistance to hypomethylating agents (HMAs), poor responses to chemotherapy, and rapid disease progression.^18,19^ These findings support the growing consensus that TP53 status, particularly its allelic state and clonal burden, should be used to stratify patients in clinical trials and guide therapeutic choices.

In contrast, SF3B1 mutations, found in up to 80% of MDS cases with ring sideroblasts, are associated with a favorable prognosis, longer leukemia-free survival, and robust erythroid responses to luspatercept, a first-in-class agent targeting the TGF-β superfamily pathway.^20,21^ These mutations are now recognized in both the 2022 WHO classification and the International Consensus Classification (ICC) as defining a distinct lower-risk MDS subtype.^22^ Clinical trial data from the MEDALIST and COMMANDS studies demonstrated that SF3B1-mutated patients had significantly higher rates of transfusion independence and durable hemoglobin increases with luspatercept therapy compared to wild-type cases.^23^ Mutations in TET2, one of the most frequently mutated genes in MDS and clonal hematopoiesis, have also been associated with increased response to azacitidine. Patients with TET2 mutations, particularly in the absence of ASXL1, had significantly higher hematologic response rates (71% versus 37%; p = 0.01) and better overall outcomes when treated with HMAs.^24^ This sensitivity may relate to TET2’s role in epigenetic regulation and promoter demethylation, facilitating reactivation of silenced tumor suppressor genes under azacitidine exposure. Conversely, co-mutations in ASXL1, RUNX1, or NRAS can attenuate this response, highlighting the importance of composite mutational profiling.^25^

Additional emerging biomarkers under investigation include SRSF2 and U2AF1 mutations, both spliceosome gene alterations that may predict resistance to HMA monotherapy and have been linked to clonal persistence after therapy. High VAFs in these genes often correlate with subclonal stability, suggesting limited responsiveness to single-agent treatments but potential vulnerability to splicing modulators or combination regimens.^26^ Incorporating these mutational markers into dynamic risk models such as IPSS-M has already improved stratification and individualization of therapy. Moreover, the advent of real-time NGS and MRD tracking is beginning to enable clinicians to adjust therapy based on evolving molecular profiles rather than static baseline characteristics. As a result, the routine inclusion of molecular biomarkers such as TP53, SF3B1, TET2, and spliceosome gene mutations in diagnostic and prognostic algorithms is rapidly becoming standard practice. These markers not only provide key prognostic information but increasingly determine eligibility for targeted agents (like eprenetapopt for TP53-mutant MDS; luspatercept for SF3B1-mutant MDS) and guide rational therapeutic combinations. The integration of biomarker-based decision-making represents a cornerstone of precision oncology in MDS.

## 3. Breakthroughs in Lower-Risk MDS Treatment

Recent advancements in the treatment of lower-risk myelodysplastic syndromes (LR-MDS) have significantly improved patient outcomes, bringing renewed hope to a population previously limited to largely supportive care. Given the heterogeneous nature of LR-MDS—often characterized by anemia, neutropenia, and thrombocytopenia—these breakthroughs are particularly impactful, as they target the underlying disease process rather than just mitigating symptoms.

Historically, treatment for LR-MDS focused on supportive interventions such as erythropoiesis-stimulating agents (ESAs) and transfusions, with curative hematopoietic stem cell transplantation largely reserved for high-risk patients. The approval of luspatercept, a first-in-class agent targeting the TGF-β pathway, marked a major shift in therapeutic strategy. The MEDALIST phase 3 trial demonstrated that luspatercept significantly improved red blood cell (RBC) transfusion independence (TI) in patients with LR-MDS, a milestone given the burden of chronic transfusions and iron overload in this population.^27^

Building on this success, the COMMANDS trial compared luspatercept to epoetin alfa, a widely used ESA, and found that luspatercept yielded superior rates of RBC TI and hemoglobin improvement. These findings firmly positioned luspatercept as a more effective option, redefining the standard of care for managing anemia in LR-MDS.^28^

Another promising agent, imetelstat, a telomerase inhibitor, is under investigation for patients with LR-MDS who have failed prior therapies. The IMerge phase 3 trial reported that imetelstat led to significant and durable improvements in hemoglobin levels, with a subset of patients achieving long-term RBC TI.^29^ These results suggest that imetelstat could offer a valuable alternative for patients with limited treatment options, particularly those refractory to ESAs.

Collectively, these studies underscore a paradigm shift in the management of LR-MDS, moving from symptomatic treatment to targeted, disease-modifying therapies. As agents like luspatercept and imetelstat gain traction, they offer not only improved efficacy but also the potential to enhance quality of life and reduce the burden of chronic transfusions. Ongoing research and real-world data will be critical to further establish their role and optimize their use in clinical practice. Continued innovation in this space holds the promise of expanding therapeutic horizons for patients with LR-MDS and improving long-term outcomes.

## 4. Limited Progress in High-Risk MDS and the Promise of Immune Checkpoint Inhibitors

### Current Standard of Care

Over the past two decades, treatment strategies for high-risk myelodysplastic syndromes (HR-MDS) have remained largely unchanged, with allogeneic HSCT remaining the only potentially curative option for eligible patients.^30^ While HMAs such as azacitidine and decitabine are widely used, they present several limitations—including modest remission rates, unpredictable patient responses, and the requirement for multiple treatment cycles. Real-world evidence has further confirmed that HMAs are not curative, reinforcing the central role of transplant in HR-MDS management.^31^

### Limitations of HMAs

Despite their approval, HMAs have not substantially improved OS in HR-MDS.^32,33^ Combination strategies—such as HMA with lenalidomide, vorinostat, or magrolimab—have been explored, but none have yet demonstrated a survival benefit sufficient to change standard practice. One promising regimen involves venetoclax plus azacitidine, which is being evaluated as frontline therapy for HR-MDS in a single-arm phase 2 trial encompassing a broad genetic spectrum.^34^

### Emerging Strategies

Venetoclax combined with azacitidine is also under investigation for relapsed/refractory MDS, where it has shown encouraging rates of transfusion independence and clinical responses.^35^ The randomized phase 3 VERONA trial is currently underway, comparing azacitidine plus venetoclax versus azacitidine plus placebo, with complete response (CR) and OS as primary endpoints.

Another promising area of exploration involves targeting immune dysregulation, a hallmark of HR-MDS, that involves impaired T-cell, NK-cell, and macrophage function.^36^ These immunologic defects have spurred interest in immune checkpoint inhibitors (ICIs); however, the complex immunosuppressive microenvironment of HR-MDS has limited their success compared to solid tumors and other hematologic malignancies.

A multicenter Phase 1/1b trial of ipilimumab in relapsed hematologic malignancies HSCT demonstrated limited efficacy in HR-MDS patients.^37^ In another study evaluating ipilimumab in the HMA-refractory setting, marrow complete response (mCR) was achieved in one patient (3.4%), and prolonged stable disease (≥46 weeks) occurred in seven patients (24%).^38^ Notably, five patients were able to undergo allo-HSCT without excess toxicity. Responders exhibited higher frequencies of Inducible Co-Stimulator ( ICOS)-expressing T cells, though increased T-cell receptor diversity did not correlate with clinical outcomes. These findings suggest potential biomarkers for benefit, but underscore the limited efficacy of ipilimumab monotherapy.

Further randomized trials combining durvalumab with azacitidine failed to show improvement in overall response rates or progression-free survival.^39^ The lack of clinical activity was not attributable to altered azacitidine exposure, changes in global DNA methylation, or immune toxicity. The exact cause however remains sketchy, some hypothesis such as compensatory programmed death ligand 2 (PD-L2) upregulation or insufficient T-cell activation were proposed but not substantiated by trial data, as no significant immune activation was observed in blood or bone marrow, despite PD-1 blockade.

Attempts to identify responsive subpopulations, such as those with crmutations or splicing factor mutations, have also yielded disappointing results. While biologically plausible, these molecular subsets have not shown clear improvements in response to ICIs in either HR-MDS or TP53-mutant AML.^40,41^

### Challenges and Future Directions

Given the limitations of current therapies, alternative strategies are under exploration. One such avenue involves entinostat, a class I histone deacetylase inhibitor (HDACi), supported by preclinical findings (Tae Kon Kim, unpublished) and under clinical investigation.^42^ A 2014 study of vorinostat plus azacitidine demonstrated a 64% overall response rate (ORR) in a phase 1 setting.^43^ In a subsequent phase 2 trial enrolling HR-MDS and AML patients with poor performance status, the ORR was 30%, with 80% surviving beyond 60 days.

Another promising agent is oral rigosertib, which showed a 90% ORR and a 34% CR rate when combined with azacitidine in a 2019 first-line trial involving HMA-naïve HR-MDS patients.^44^ These results compare favorably to historical CR rates (<20%) with azacitidine monotherapy. Importantly, 30% of patients achieved transfusion independence—a clinically meaningful outcome that merits further validation in a larger, randomized phase 3 trial. The regimen was also well tolerated, underscoring its potential viability in frail or elderly patient populations.

## 5. Future Directions in MDS: Beyond Current Limitations

Despite recent therapeutic advancements, high-risk myelodysplastic syndromes (HR-MDS) remain a formidable clinical challenge with poor long-term outcomes. While HMAs remain the standard of care, they confer only modest survival benefits, and resistance is common, often culminating in disease progression. Allogeneic HSCT remains the only potentially curative option for patients with HR-MDS.^45^

Given these limitations, novel biologic agents are being actively investigated to target critical pathways involved in MDS pathogenesis.

### Immunotherapies and Targeted Agents

Sabatolimab, a humanized monoclonal antibody against T-cell immunoglobulin and mucin domain-containing protein 3 (TIM-3)—a key regulator of immune responses and leukemia progression—has emerged as a promising immunotherapeutic candidate.^46^ In the STIMULUS-MDS1 phase II trial, sabatolimab combined with HMA demonstrated an acceptable safety profile in patients with intermediate to very high-risk MDS, but failed to significantly improve CR rates or PFS.^47,48^ Despite this, the favourable safety data supported its advancement to the STIMULUS-MDS2 phase III trial, which included patients with CMML to assess its effect on OS.^49^ However, the trial was suspended in January 2024 due to failure to meet its primary endpoint. TIM-3 co-expression with PD-1 and LAG-3 and the ‘cold’ MDS marrow microenvironment may limit monotherapy efficacy in the absence of predictive biomarkers.^50,51^

Pevonedistat, a selective inhibitor of the Neural Precursor Cell Expressed Developmentally Downgraded 8 (NEDD8-activating enzyme) critical for ubiquitination and protein degradation, initially demonstrated promise when combined with azacitidine, improving overall survival (OS), event-free survival (EFS), and response duration in early-phase studies.^52–54^ However, the phase III PANTHER trial failed to show a significant difference in median EFS or OS, contrasting earlier findings.^55^ Pevonedistat’s cytostatic rather than cytotoxic mechanism and non-selective hematopoietic suppression, combined with a lack of biomarker-driven selection during trials, likely contributed to its limited clinical efficacy.^56^

Eprenetapopt, a first-in-class small molecule that restores wild-type p53 function in TP53-mutant cells, has shown encouraging results in early trials.^57,58^ A phase Ib/II study combining eprenetapopt with azacitidine reported promising CR rates in TP53-mutant MDS. Nevertheless, febrile neutropenia occurred in one-third of patients, raising safety concerns despite the agent’s efficacy.^58^ APR-246’s p53-independent oxidative stress and unstratified TP53 mutational heterogeneity likely contributed to the off-target toxicity and variable responses.^59^

### Therapies with Limited Clinical Impact

Several agents that showed early promise failed to demonstrate clinical benefit in later trials. Glasdegib, an oral inhibitor of the Hedgehog signaling pathway, combined with low-dose cytarabine, achieved only a 20% response rate and was associated with a high discontinuation rate due to toxicity.^60^ Glasdegib’s limited efficacy may reflect pathway cross-talk with SDF-1/CXCR4 signaling and absence of predictive baseline mutations, highlighting the need for biomarker-driven strategies targeting LSC-associated resistance mechanisms.^61^ Rigosertib, a renin-angiotensin system (RAS) pathway inhibitor, failed to improve OS in a phase III trial,^62^ although a more recent phase I/II study combining it with azacitidine showed improved ORR, suggesting potential synergy that warrants further investigation.^63^ Rigosertib likely failed due to an unclear mechanism and lack of biomarker-based selection (marrow blast percentage), despite early signs of synergy with azacitidine.^64^ Magrolimab, a CD47-targeting monoclonal antibody, demonstrated an impressive 75% ORR and 33% CR rate when combined with azacitidine in TP53-mutated MDS,^65^ but the phase III ENHANCE trial was ultimately discontinued due to futility.^66^ CD47’s expression on erythrocytes led to anemia and hemolytic toxicity, while TAM-driven immunosuppression and adaptive resistance likely limited magrolimab’s long-term efficacy in MDS.^67^

Tamibarotene (SY-1425), a selective retinoic acid receptor alpha (RARα) agonist, demonstrated improved clinical outcomes in RARα-positive HR-MDS when combined with azacitidine in early trials.^68,69^ However, the SELECT-MDS-1 phase III trial was discontinued in November 2024 after failing to meet its primary endpoint.^70^ Tamibarotene likely failed due to variable or transient RARα expression and limited patient stratification, undermining its efficacy in an unselected HR-MDS population.^71^

These failures highlight the need for mechanism-aligned trial design—matching to oncogenic drivers or immune escape pathways, integrating molecular stratification (e.g., TIM-3⁺, TP53 multi-hit), and incorporating dynamic pharmacodynamic biomarkers.

### Immune Checkpoint Inhibitors

Checkpoint inhibitors have also been evaluated in MDS. PD-1 inhibitors, such as pembrolizumab and nivolumab, showed some efficacy in newly diagnosed HR-MDS, but their benefit was limited in HMA-refractory settings.^72–74^ PD-L1 inhibition with durvalumab resulted in improved ORR but was constrained by notable toxicity.^75^ These findings suggest that combination strategies may be required to fully realize the potential of checkpoint blockade in HR-MDS.

CTLA-4 blockade with ipilimumab demonstrated a 21% clinical benefit rate in a phase Ib trial for HMA-refractory HR-MDS.^76,77^ A subsequent phase II study combining ipilimumab with azacitidine achieved an ORR of 71% (compared to 35% with ipilimumab alone), supporting further evaluation in larger studies.^78^

### Emerging Therapeutic Strategies

While numerous agents have failed to achieve their primary endpoints in late-phase trials, ongoing efforts are exploring innovative therapeutic modalities, including kinase inhibitors, immune-based strategies, and adoptive cell therapies aimed at overcoming resistance and improving outcomes.

Bemcentinib, a selective AXL receptor tyrosine kinase inhibitor, was evaluated in the BERMAGO phase II trial for refractory HR-MDS. Despite a favorable safety profile, limited clinical efficacy led to early termination due to disease progression (NCT03824080).^79^ Emavusertib, an interleukin-1 receptor-associated kinase 4 (IRAK4) inhibitor involved in Toll-like receptor and interleukin-1 receptor (IL-1R) signaling, is under evaluation in combination with venetoclax for HR-MDS and AML (NCT0427876800). Tomaralimab, a monoclonal antibody targeting Toll likereceptor 2 (TLR2), overexpressed in CD34+ cells in low-risk MDS, has shown potential for patients with HMA-refractory LR-MDS.^80^

### Adoptive Cell Therapies

Adoptive T-cell therapies, including chimeric antigen receptor T-cell therapy (CAR-T), tumor infiltrating lymphocytes (TIL), and T-cell receptor T-cell therapy (TCR-T), have revolutionized lymphoma treatment, but remain underexplored in myeloid malignancies. CAR-T therapies targeting NKG2D (NCT02203825) and CD123 show early promise in HR-MDS.^77^

Dendritic cell (DC)-based immunotherapies, such as MoDC and DCleu vaccines, enhance antigen presentation and leukemia-specific T-cell responses, offering potential therapeutic benefit in HR-MDS.^81,82^

Similarly, natural killer (NK) cell adoptive transfer has shown anti-tumor activity in HR-MDS. Preclinical and early *in vivo* studies suggest NK cell infusion may serve as a bridge to allogeneic HSCT, though further investigation is required to optimize combinations and improve efficacy.^83^

While significant obstacles remain in HR-MDS treatment, ongoing development of targeted agents, immunotherapies, and cellular strategies continues to offer hope for transformative therapies and improved patient outcomes.

## Conclusion

The therapeutic landscape of MDS has evolved significantly over the past decade, yet major challenges persist, particularly in the treatment of HR-MDS with poor prognoses and limited responses to current therapies. While hypomethylating agents remain the cornerstone of treatment, their transient efficacy highlights the need for more effective and personalized approaches.

The emergence of novel agents including luspatercept and imetelstat have redefined care in lower-risk MDS, while promising novel strategies—including p53 reactivators, CD47 inhibitors, T-cell TIM-3 blockers, and immune checkpoint inhibitors—are reshaping the treatment paradigm in higher-risk disease. Despite early successes, many investigational agents have failed to demonstrate sustained clinical benefit in late-phase trials, underscoring the complexity of MDS pathogenesis and resistance mechanisms. However, the emergence of advanced molecular profiling, risk-adapted treatment strategies, and combination approaches has laid the foundation for a more precise and dynamic therapeutic framework.

A major limitation in the current treatment approach lies in the clinical trial design, where molecular heterogeneity is often overlooked. Many studies continue to apply the same treatment models to biologically diverse patient populations, limiting the potential to identify effective personalized therapies. Moreover, the absence of dynamic tools such as MRD monitoring prevents timely treatment adaptation and prediction of possible relapses.

As our understanding of the genetic, immunologic, and microenvironmental drivers of MDS continues to deepen, the integration of novel agents—combined with predictive biomarkers and innovative trial designs—will be critical in advancing personalized care. Future research must focus on real-time molecular testing to guide therapy tailored to patients’ individual risk, and rational combination therapies that target both the primary disease drivers and the tumor microenvironment. Importance must be given to the development of adaptive clinical trial designs, exploring early intervention strategies in patients with clonal cytopenias, and emphasizing on quality of life outcomes alongside survival metrics.

Ultimately, while challenges remain, the convergence of translational science, clinical innovation and patient-centered research offers renewed hope for achieving durable remissions and improved long-term outcomes in patients with MDS.

### Author Contributions (CRediT Taxonomy)

Conceptualization: Viviana Cortiana, Harshitha Vallabhaneni, Yan Leyfman

Methodology: Viviana Cortiana, Harshitha Vallabhaneni, Kennedy Itodo

Formal Analysis: Jenna Ghazal, Taha Kassim Dohadwala

Investigation: Viviana Cortiana, Harshitha Vallabhaneni, Jenna Ghazal

Writing – Original Draft Preparation: Viviana Cortiana, Harshitha Vallabhaneni, Jenna Ghazal

Writing – Review & Editing: Yan Leyfman, Chandler H. Park, Kennedy Itodo

Resources: Yan Leyfman, Chandler H. Park

Supervision: Yan Leyfman, Chandler H. Park

Visualization: Jenna Ghazal, Taha Kassim Dohadwala, Harshitha Vallabhaneni

Project Administration: Viviana Cortiana

### Institutional Review Board Statement

Not applicable.

### Informed Consent Statement

Not applicable.

### Conflicts of Interest

The authors declare no conflicts of interest.

## Data Availability

No patient data was directly utilized in this study.
